# Deflection Intelligent Prediction for High-Strength Steel Saddle Plate Forming Applicable to Reducing Ship Weight

**DOI:** 10.3390/ma16176028

**Published:** 2023-09-01

**Authors:** Shun Wang, Jinliang Dai, Zhikang Xu, Ji Wang, Rui Li, Jiayan Wang

**Affiliations:** 1 Naval Architecture and Ocean Engineering College, Dalian Maritime University, Dalian 116026, China; daijinliang@dlmu.edu.cn (J.D.); xuzhikang@dlmu.edu.cn (Z.X.); wangjiayan@dlmu.edu.cn (J.W.); 2 School of Naval Architecture and Ocean Engineering, Dalian University of Technology, Dalian 116024, China; wangji@dlut.edu.cn (J.W.); lirui@dlut.edu.cn (R.L.)

**Keywords:** plate forming, ship lightweight, line heating, high-strength steel, deflection prediction, support vector machine

## Abstract

The application of high-strength steel plates can reduce ship weight, and the saddle plate is one of the most common types of double-curved hull plates. To fill the research gap regarding high-strength steel saddle plates, two prediction models are established here to predict deformation in saddle plate forming. Deflection is a key parameter reflecting the overall deformation of a curved plate. Therefore, first of all, the influencing factors of the line heating of high-strength steel saddle plates were analyzed. The influence of plate geometric parameters and forming parameters on deflection was researched. Second, a multiple linear regression model between deflection and the geometric parameters and forming parameters of high-strength steel saddle plates was established. Finally, to solve the problem of a large error in the multivariate regression model for extrapolation, an intelligent prediction program for deflection based on a support vector machine (SVM) was developed using the Python language. The results show that the error of the multiple regression model was less than 5% for data interpolation. The error of the intelligent prediction model for deflection was less than 5% for data extrapolation. This research can provide data support for the automatic forming of marine saddle plates.

## 1. Introduction

In recent years, global warming has accelerated significantly, resulting in accelerated melting of the Arctic Ocean’s ice. Once the Arctic Channel is fully opened, it will directly change the maritime transportation structure. There will be a significant impact on international trade and energy development. However, there is a higher strength requirement for hull plates for polar navigation. So, higher-strength materials are needed for hull plates. In addition, the Initial IMO Strategy for the reduction of greenhouse gas (GHG) emissions from ships was proposed by the International Maritime Organization (IMO) in 2018. The average CO_2_ emissions of international shipping will be reduced by at least 40% compared with 2008 and reduced by 70% by 2050 via this strategy [1]. The lightweight construction of ships is one of the ways to achieve CO_2_ emission reduction. On the premise of ensuring sufficient strength, the thickness of high-strength steel is lower than that of traditional ordinary steel plates. The weight of an empty ship can be reduced effectively by applying high-strength steel, which not only reduces the emissions of CO_2_ but also reduces the transportation cost [2]. High-strength steel is becoming a hot research topic regarding ship structural materials due to polar and green navigation. In order to research the influence of the low-temperature Arctic environment on the properties of high-strength steel materials, Chen, M. et al. [3] carried out tensile tests on steel Q890 and steel Q960 in a liquid nitrogen chamber. The mechanical properties, such as the yield stress of high-strength steel at low Arctic temperatures, were obtained, and the prediction equation was proposed. Li, J. et al. [4] researched the deformation mechanism of AH36 L-beams under air cooling by numerical simulation. The deformation of L-beams is caused by the combined action of the transformation strain and the cooling shrinkage strain. Netto, A. et al. [5] established a numerical calculation model for the welding of high-strength steel and optimized the process parameters. Obers, S. et al. [6] proposed a finite element simulation model for nonlinear plastic materials to analyze the effect of the yield-to-tensile strength ratio on the plastic stress concentration factor. Ramírez-Ramírez, J. et al. [7] analyzed the causes of cracks in high-strength steel and evaluated these causes through numerical calculation models. Sorger, G. et al. [8] studied the fatigue performance of high-strength steel in welding and concluded that the fatigue strength of welded joints in friction stir welding was higher than that in electric arc welding. Most of the existing studies on high-strength steel have focused on welding, material properties, and fatigue strength. However, few scholars have paid attention to the study of the plate forming of high-strength steel.

The hull plate is an important part of the ship structure, in which a large number of double-curved hull plates are used to form the complex shape of the bow and stern to meet the hydrodynamic requirements of the ship. At present, the main method of double-curved hull plate forming is line heating [9]. The equipment cost of line heating is relatively low, which has high economic benefits in the process of ship construction. However, at present, the automation degree of line heating is not high. Dependence on the experience of workers is strong [10]. Therefore, many scholars have performed further research on the mechanism of line heating. Vega, A. et al. [11] researched the effect of overlapping heating on the inherent strain in line heating by finite element analysis. The results showed that the inherent strain was caused by the residual stress generated by the previous heating line. Focusing on the line heating of a single-curved plate, Yona, D. et al. [12] researched the influence of plate thickness, heating times, and heating velocity on deformation by combining numerical calculation and experimentation. It was considered that plate thickness was the most important factor affecting deformation. Shibahara M et al. [13] combined the finite element method with deep reinforcement learning to obtain process plans for line heating. Thomas K. et al. [14] established a computer simulation model for line heating, which could predict the deformation of flat plates. Lim H. et al. [15] studied the effect of line heating on the material properties of steel plates, and the experimental results showed that the steel plates formed by line heating had greater yield strength and tensile strength. Wang, J. et al. [16] carried out induction heating experiments on steel sail plate Q235 and steel saddle plate AH36. A numerical calculation model of induction heating in curved plate forming was established, which matched the experimental results. Zhou, H. et al. [17] researched the influence of heating velocity on the maximum temperature of the plate surface in induction heating and proved the feasibility of numerical calculation using elastic finite element analysis combined with experiments. Zhang, S. et al. [18] optimized the shape parameters of the inductor in induction heating and found that the deformation of the curved plate significantly increased through optimization. Wang, S. et al. [19] established a numerical calculation model for high-strength steel saddle plates through experiments and studied the influence of geometric and process parameters on the deformation of the plates. Lee, J. et al. [20] derived a formula for calculating the transverse shrinkage and angular deformation of curved plates by using plate thickness, heating velocity, and heating line spacing based on the results of multi-heating line forming experiments and numerical analysis. The formula could also be used to calculate the minimum distance between two heating lines that do not interact with each other. Zhao, Z. et al. [21] researched the influence of forming parameters on plastic strain in induction heating and established a database of the relationship between forming parameters and plastic strain under a single heating line condition. At present, scholars’ research on line heating has mainly been about low-carbon steel materials, and the types of plates studied have mainly been sail-shaped plates and single-curved plates. Therefore, there is a lack of research on the forming of high-strength steel saddle plates.

The saddle plate is one of the most common types of double-curved hull plate, which is applied to the outer hull to reduce navigation resistance. Deflection is an important parameter to express the overall deformation of the saddle plate. To fill the research gap in the forming of high-strength steel saddle plates, the intelligent prediction model is established in this paper based on the numerical calculation model of high-strength steel saddle plates in previous research [19]. Firstly, the influence of forming parameters and plate geometric parameters of the plate on the deflection is researched. Secondly, a multiple regression deflection prediction model of high-strength steel saddle plate suitable for data interpolation is established. Finally, in order to realize the deflection prediction during data extrapolation, an intelligent prediction model for deflection based on a support vector machine (SVM) is established. The deflection can be quickly calculated by inputting geometric parameters and forming parameters into the model. The research in this paper belongs to the application of artificial intelligence in the shipbuilding industry, which can provide data support for the automatic forming of curved plates in the fields of chemistry, aerospace, and so on.

## 2. Deformation Influence Law of High-Strength Steel Saddle Plate

**Figure 1:**
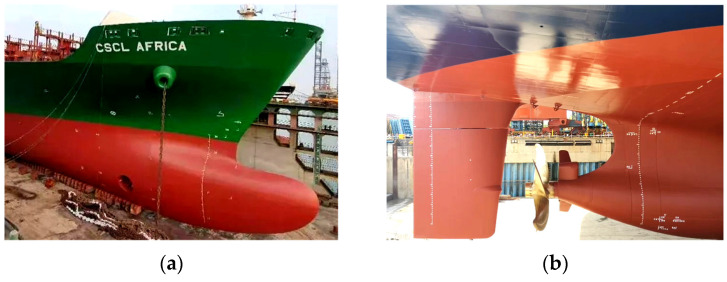
(**a**) The shape of bow; (**b**) the shape of stern.

**Figure 2:**
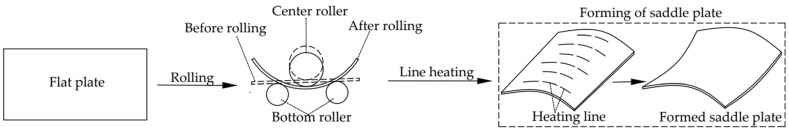
Saddle plate forming process.

**Figure 3:**
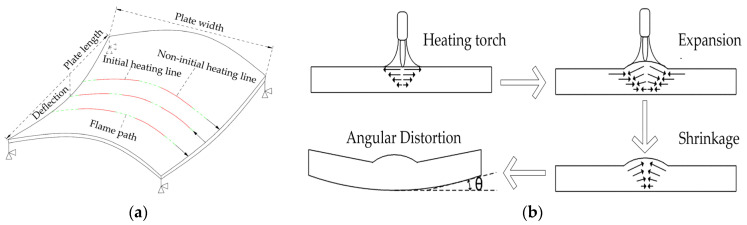
(**a**) Forming characteristics of line heating for saddle plate; (**b**) line heating mechanism.

As shown in Figure 1, the shape of the bow and stern is steeper than that of the parallel middle body in the middle of the hull. A large number of double-curved plates are applied to meet the complex shape demands of the hull. As one of the most common types of double-curved hull plates, the forming quality of saddle plates affects the quality of hull plates. As shown in Figure 2, the forming of the saddle plate is divided into two stages. Firstly, the plate is formed into a single-curved plate with a certain curvature by means of a roller bed. Then, the curvature in the other direction is obtained during the line heating process. The forming characteristics of line heating for saddle plates are shown in Figure 3a. A uniaxial support is set on the four corners of the plate to suspend the plate. The heating line is located on the convex surface of the single-curved plate. The heat source moves uniformly along the heating line, while the cooling water moves uniformly with the heat source. These two keep a certain distance. The area near the heating line is first heated and then cooled. The shrinkage is greater than the expansion, and the curved plate thus produces irreversible bending deformation, as shown in Figure 3b. The convex surface of the single-curved hull plate produces a vertical downward deformation, which makes the plate deform into a saddle shape. The deflection is defined as the vertical displacement along plate length, which reflects the overall deformation of the curved plate.

**Figure 4:**
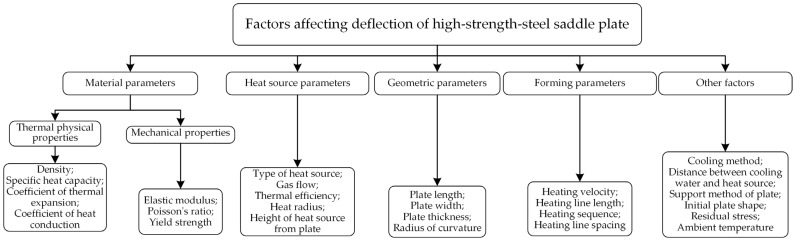
Influencing factors of deflection of high-strength steel saddle plate.

**Figure 5:**
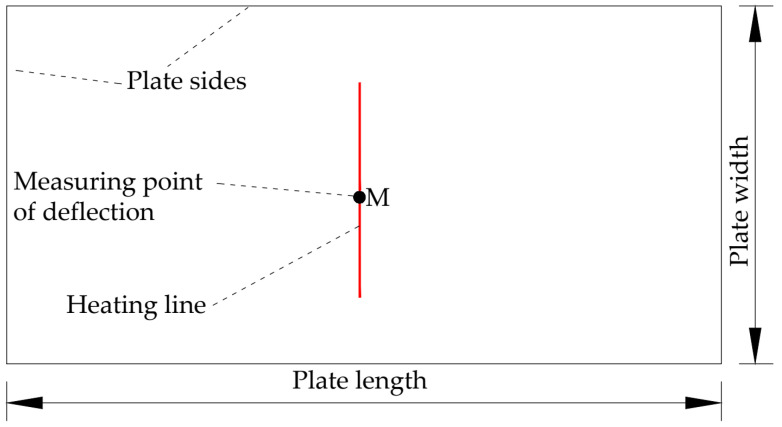
Location of heating line and deflection monitoring points.

As shown in Figure 4, the deflection of the saddle plate is influenced by different types of factors. The type of high-strength steel selected is EH36, and its chemical composition is shown in Table 1. The propylene heat source in a certain shipyard is selected as the heat source type. The cooling water is applied to the heated surface. The cooling water is located at a distance of 0.15 m behind the heat source. The initial shape of the plate is a single-curved plate with unidirectional support at the four corners. The ambient temperature is set as 20 °C. Therefore, material parameters, heat source parameters, and other factors are determined. The influence of geometric parameters and forming parameters on deflection needs to be studied. In the research of the influence law of high-strength steel saddle plate forming, the basic parameters of the finite element model of a plate are set as follows: The plate length is 3 m. The plate width is 1.5 m. The curvature radius is 5 m. The plate thickness is 0.012 m. The heating line is set in the middle of the plate, whose length is 0.3 m, as shown in Figure 5. No special instructions in the following are set according to the basic parameters. Since the center point M of the plate can best reflect the overall deformation, this point is selected as the measuring point of deflection.

### 2.1. The Influence of Forming Parameters on the Deflection of High-Strength Steel Saddle Plate

In the previous research [19], a numerical calculation model for the forming of high-strength steel saddle plates has been established, and the feasibility of this model has been verified through experiments. Therefore, without special instructions, the results of deflection and temperature in the following text are obtained through a numerical calculation model. In the design of independent variables, the forming parameters (heating velocity and heating line length) are set based on the processing experience, and the geometric parameters (plate length, plate width, plate thickness, and curvature radius) of the plate are set based on the size of the real hull plate. Based on the above explanation, the forming influence laws of high-strength steel saddle plates are researched.

#### 2.1.1. The Influence of Heating Velocity on the Deflection of High-Strength Steel Saddle Plate

**Figure 6:**
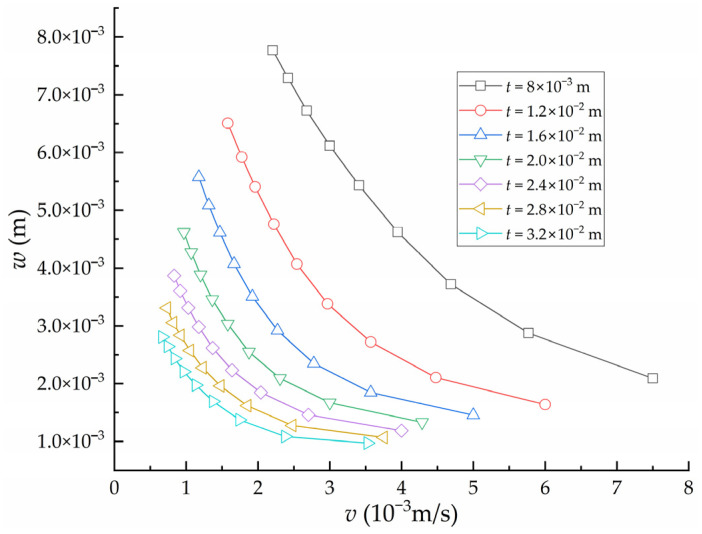
The influence of heating velocity on the deflection of high-strength steel saddle plate.

**Figure 7:**
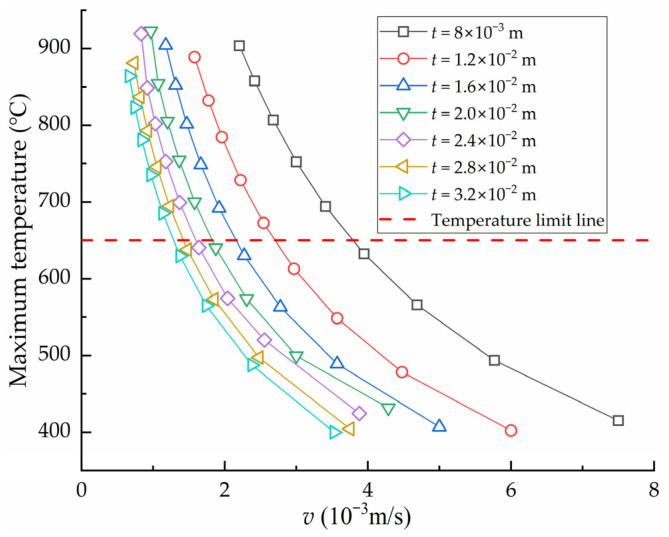
The influence of heating velocity on maximum temperature of high-strength steel saddle plate.

Keeping other parameters except the heating velocity *v* constant, the influence of the heating velocity on the deflection *w* of high-strength steel saddle plate under different plate thickness *t* is obtained, as shown in Figure 6. Under the condition of the same plate thickness, the smaller the heating velocity, the greater the deflection. However, according to forming experience, the surface temperature of the plate is negatively correlated with the heating velocity. If the surface temperature of the plate is too high, the material quality of the plate may be destroyed. Then, the plate performance cannot meet the engineering requirements of shipbuilding. Therefore, the maximum temperature of the plate surface during the forming process of the high-strength steel saddle plate is an important constraint on the heating velocity. Keeping other parameters except the heating velocity *v* constant, the influence of the heating velocity of series plate thickness on the maximum temperature of the plate surface is obtained, as shown in Figure 7. The surface temperature of the plate increases rapidly with the decrease in the heating velocity under the same plate thickness. At the same heating velocity, the surface maximum temperature decreases with the increase in plate thickness. The greater the plate thickness, the more heat is absorbed by cold metal in the thickness direction, resulting in a decrease in temperature.

**Figure 8:**
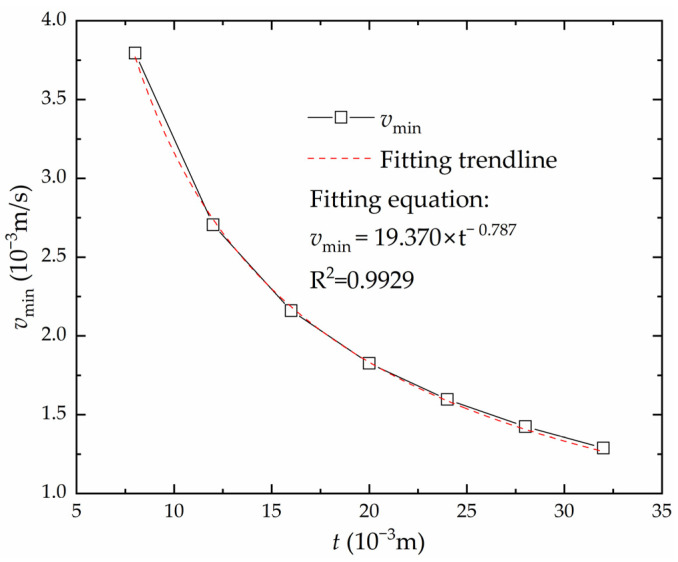
The relationship between plate thickness and minimum heating velocity.

According to the China Shipbuilding Quality Standard and forming experience, when the cooling method is water cooling, the maximum temperature of high-strength steel EH36 should be lower than 650 °C [22]. This temperature is marked in Figure 7, which is called the temperature limit line. The velocity that corresponds to the intersection of the temperature limit line and the curve is defined as the minimum heating velocity $v_{min}$, which is the velocity that maximizes the forming deflection without destroying the steel plate material. In order to improve the forming efficiency, the number of heating lines needs to be reduced. The smaller the heating velocity, the greater the deflection generated by a single heating line, and the fewer heating lines are required to produce the same deformation. The relationship curve between the plate thickness and the minimum heating velocity $v_{min}$ is shown in Figure 8. The greater the plate thickness, the smaller the minimum heating velocity of the saddle plate forming. In order to quickly calculate the minimum heating velocity for different plate thicknesses, the curve is nonlinearly fitted as follows:

(1) $$v_{min}=19.370\times t^{-0.787}$$

The goodness of fit of R^2^ is 0.9929, which indicates that the fitting degree is good.

The minimum heating velocity under different plate thicknesses is calculated by Equation (1). The maximum temperature at the minimum heating velocity is obtained by numerical calculation. As shown in Table 2, the maximum relative error between the maximum temperature and 650 °C is 0.97%. The validity of Equation (1) is proven.

#### 2.1.2. The Influence Law of the Heating Line Length on the High-Strength Steel Saddle Plate

**Figure 9:**
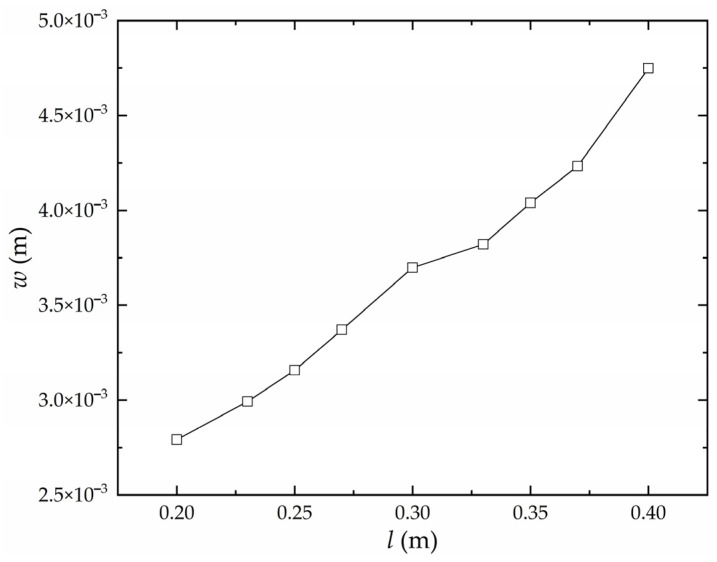
The influence of heating line on the deflection of high-strength steel saddle plate.

Keeping other factors except heating line length constant, the influence of heating line length on the deflection of the high-strength steel saddle plate is obtained, as shown in Figure 9. At the same heating velocity, the heating line length is positively correlated with the heat absorbed by the plate during the forming process. The deflection is positively correlated with the heating line length.

### 2.2. The Influence of Plate Geometric Parameters on the Deflection of High-Strength Steel Saddle Plate

Considering the curved forming parameters of a real hull plate, the plate geometric parameters are set. The range of plate width is 1 to 2.5 m. The range of plate length is 1.5 to 5.0 m. The range of plate thickness is 0.008 to 0.028 m. The range of plate curvature radius is 1.5 m to 5.0 m. Based on this, the influence of geometric parameters on the deflection is researched.

#### 2.2.1. The Influence of Plate Length, Plate Width, and Curvature Radius on the Deflection of High-Strength Steel Saddle Plate

**Figure 10:**
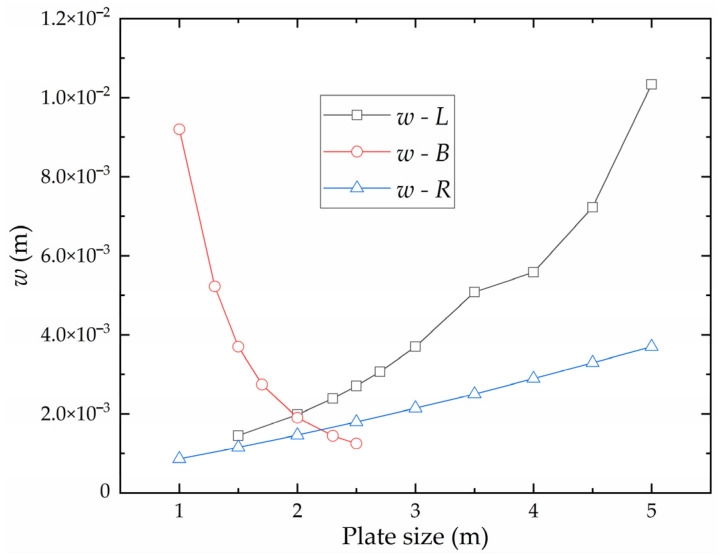
The influence of plate length, plate width, and curvature radius on the deflection of high-strength steel saddle plate.

The influence of plate length, plate width, and curvature radius on the forming deflection is shown in Figure 10. The deflection is positively correlated with plate length and negatively correlated with plate width. With the increase in plate length, the influence of plate length on deflection becomes larger. The deflection is positively correlated with the curvature radius. With the increase in curvature radius, the deflection always maintains a relatively stable sensitivity to the curvature radius.

#### 2.2.2. The Influence of Plate Thickness on the Deflection of High-Strength Steel Saddle Plate

**Figure 11:**
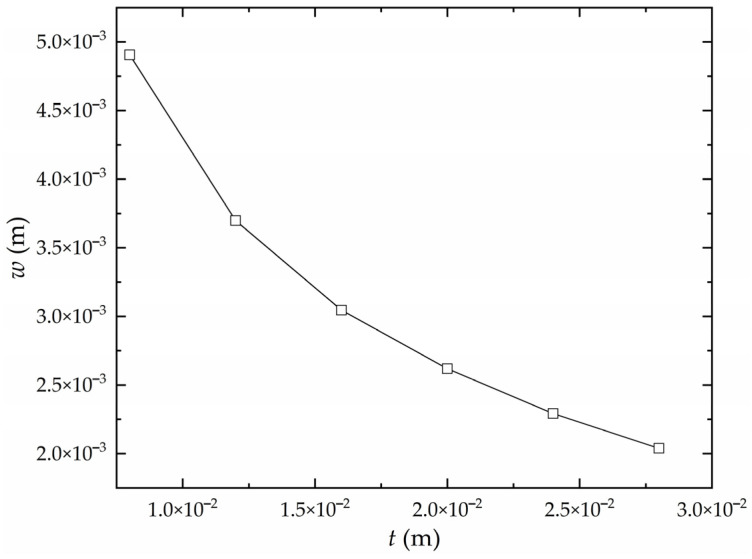
The influence of plate thickness on the deflection of high-strength steel saddle plate.

Under the condition of minimum heating velocity, the deflection of high-strength steel saddle plates with different thicknesses is shown in Figure 11. At the same maximum temperature of the plate surface, the deflection is negatively correlated with plate thickness. Therefore, the saddle plate with a larger thickness is more difficult to form.

## 3. Deflection Prediction Model of High-Strength Steel Saddle Plate Based on Multiple Regression

From the above laws, it can be seen that the relationship between deflection and influencing factors is not all linear. In order to establish the multiple linear regression model of the relationship between deflection and influencing factors, the independent variables of the nonlinear relationship are linearized. The fitting function obtained by fitting the relationship curve between deflection and influencing factors is transformed into a linear function.

It is found that except for the linear relationship between the deflection and curvature radius and heating line length, the relationship between the deflection and other influencing factors is close to the quadratic function. Therefore, the nonlinear influencing factors can be linearly transformed by perfect square expression. The perfect square formula is applied for linear transformation due to its advantages of simple ideas and rapid modeling.

### 3.1. Linearization Transformation of Deflection and Influencing Factors

#### 3.1.1. Linear Transformation of the Relationship between Deflection and Plate Length

**Figure 12:**
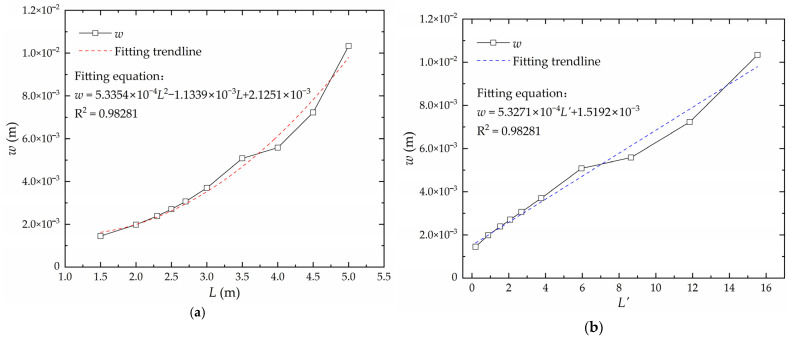
The relationship between deflection and plate length: (**a**) real plate length; (**b**) converted plate length.

Keeping other factors except plate length *L* constant, the relationship curve between deflection *w* and plate length *L* is shown in Figure 12a. Using the perfect square expression for linear transformation, there is a linear relationship between the deflection and the converted plate length *L*′, as shown in Figure 12b. The converted plate length is obtained according to Equation (2) as follows:

(2) $$L^{\prime }={\left(L-1.062\right)}^{2}$$

#### 3.1.2. Linear Transformation between Deflection and Plate Width

**Figure 13:**
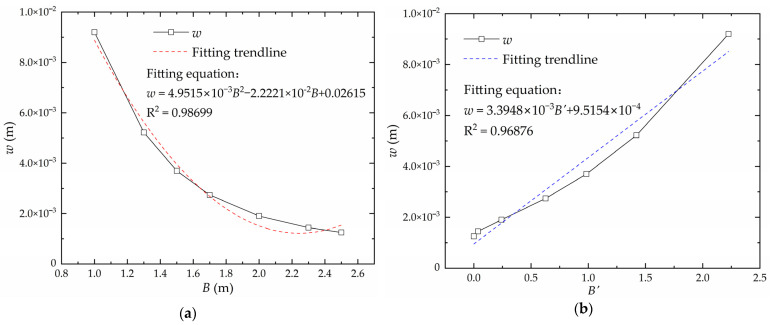
The relationship between deflection and plate width: (**a**) real plate width; (**b**) converted plate width.

Keeping other factors except plate width *B* constant, the relationship curve between deflection *w* and plate width *B* is shown in Figure 13a. Using the perfect square expression for linear transformation, there is a linear relationship between the deflection and the converted plate width $B^{\prime }$, as shown in Figure 13b. The converted plate width is obtained according to Equation (3):

(3) $$B^{\prime }={\left(B-2.244\right)}^{2}$$

#### 3.1.3. Linear Transformation of the Relationship between Deflection and Plate Thickness

**Figure 14:**
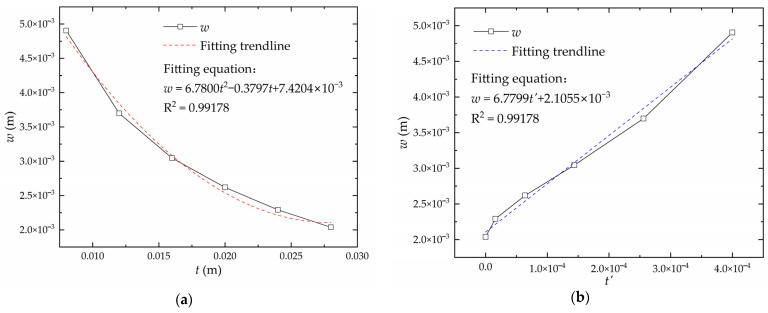
The relationship between deflection and plate length: (**a**) real plate thickness; (**b**) converted plate thickness.

Keeping other factors except plate thickness *t* constant, the relationship curve between deflection *w* and plate thickness *t* is shown in Figure 14a. Using the perfect square expression for linear transformation, there is a linear relationship between the deflection and the converted plate thickness $t^{\prime }$, as shown in Figure 14b. The converted plate thickness is obtained according to Equation (4):

(4) $$t^{\prime }={\left(t-0.027\right)}^{2}$$

#### 3.1.4. Linear Transformation of the Relationship between Deflection and Curvature Radius

**Figure 15:**
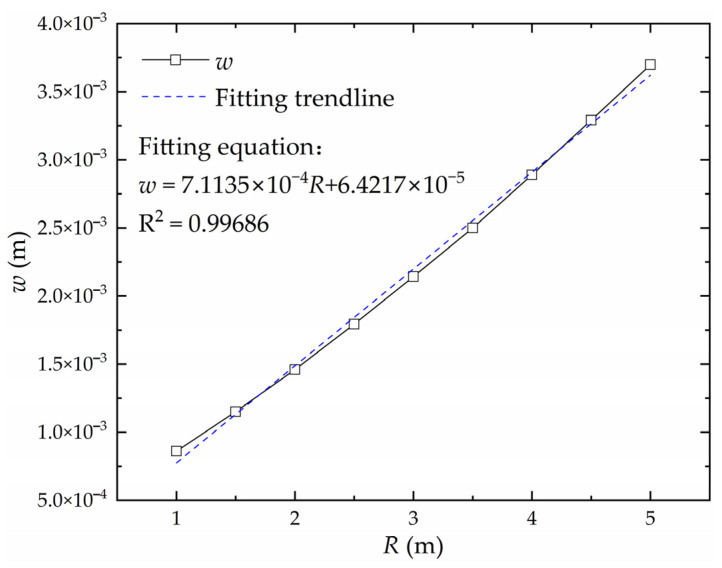
Relationship between deflection and curvature radius.

Keeping other factors except for curvature radius *R* constant, the relationship curve between deflection *w* and plate thickness *t* is shown in Figure 15. The curve is linearly fitted, and the fitting trendline satisfies the linear relationship. The relationship between the deflection and curvature radius does not need to be linearly converted.

#### 3.1.5. Linear Transformation of the Relationship between Deflection and Heating Line Length

**Figure 16:**
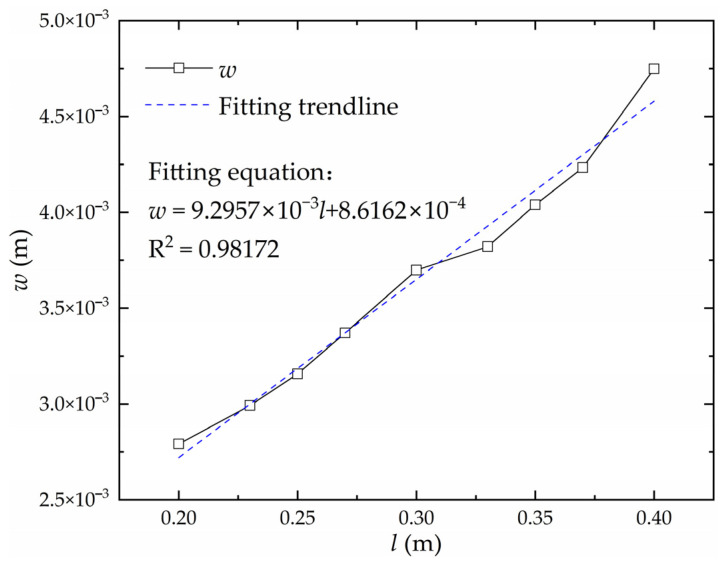
Relationship between deflection and heating line length.

Keeping other factors except heating line length *l* constant, the relationship curve between deflection *w* and plate thickness *t* is shown in Figure 16. The curve is linearly fitted, and the fitting trendline satisfies the linear relationship. The relationship between the deflection and heating line length does not need to be linearly converted.

### 3.2. Multiple Regression Model of Deflection for High-Strength Steel Saddle Plate

The prediction model of deflection is established by regression analysis. The advantages of the multiple linear regression model are as follows. The modeling velocity is fast and does not require very complex calculations. The model is more convenient for analyzing multiple influencing factors. The correlation between the different influencing factors can be accurately expressed.

There is a linear relationship between deflection and converted plate length $L^{\prime }$, converted plate width $B^{\prime }$, curvature radius *R*, converted plate length $t^{\prime },$ and heating line length *l.* Therefore, the deflection *w* can be obtained by the following multiple regression Equation (5):

(5) $$w=\beta_{0}+\beta_{1}L^{\prime }+\beta_{2}B^{\prime }+\beta_{3}R+\beta_{4}t^{\prime }+\beta_{5}l^{\prime }$$

According to the 42 sets of data in Figure 12b, Figure 13b, Figure 14b, Figure 15, and Figure 16, the six unknowns are calculated in the statistical software by Equation (6).

(6) $$\begin{array}{c} w=-9.07\times 10^{-3}+5.23\times 10^{-10}L^{\prime }+4.89\times 10^{-6}B^{\prime }+7.40\times 10^{-7}R\\ +6.31\times 10^{-9}t^{\prime }+9.30\times 10^{-6}l^{\prime } \end{array}$$

In order to test the significance of the linear relationship between the predictive variables and the controllable variables, the significance test of the regression equation is carried out. Under the condition that the significance level *α* is 0.01, the *F* test method is used to test the significance of the linear regression model. The hypothesis testing Equation (7) is as follows:

(7) $$H_{0}:\beta_{0}=\beta_{1}=\beta_{2}=\beta_{3}=\beta_{4}=\beta_{5}=0$$

If the hypothesis is rejected, it is proven that the model is significant. The calculation Equation (8) of test statistic *F* is as follows:

(8) $$F=\frac{ESS/5}{RSS/\left(42-5-1\right)}\sim F\left(5,36\right)$$

where *F* is the test statistical value; *ESS* is the regression sum of squares; and *RSS* is the residual sum of squares. Under the condition that the significance level *α* is 0.01 when $P(F>F_{0.01}\left(5,26\right))=0.01$, the critical value $F_{0.01}\left(5,36\right)$ is 3.57 by looking up the *F* distribution table. Through calculation, the value of *ESS* is $1.53\times 10^{-7}$, and the value of *RSS* is $1.53\times 10^{-7}$. Substituting *ESS* and *RSS* into Equation (8), the value of *F* is 396.52. Because the value of F is greater than $F_{0.01}\left(5,26\right)$, the hypothesis is rejected, and the prediction model is proven to be effective.

### 3.3. Example Verification of Multiple Regression Deflection Prediction Model

Six sets of examples are designed to test the accuracy of the multiple regression model. The absolute error and relative error of the prediction results are calculated. The absolute error refers to the difference between the predicted value and the value obtained by FEM, which not only shows the size of the error, but also shows the positive and negative directions, reflecting the deviation of the predicted results from the value obtained by FEM. The relative error refers to the percentage of the absolute error to the value obtained by FEM. As shown in Table 3, the maximum absolute value of absolute error is $1.52\times 10^{-4}$ m and the value of relative error is within 5%. However, when the data are extrapolated, the relative error is greater than 5%. The reliability of the multiple regression model is verified in the case of interpolation.

## 4. Intelligent Prediction Model for Deflection of High-Strength Steel Saddle Plate

When the multiple regression model is applied to the deflection prediction of the extrapolation data, the error is large. To solve this problem, an intelligent deflection prediction model of high-strength steel saddle plates based on an SVM is proposed. The five characteristic quantities of plate length, plate width, curvature radius, plate thickness, and heating line length are used as the input vectors of the intelligent prediction model. The deflection is the target vector of the prediction model. In this model, the deflection of the high-strength steel saddle plate can be predicted according to these five characteristic quantities.

### 4.1. Experimental Design of SVM

An SVM is a supervised learning algorithm that is widely applied in classification and regression problems. When the sample data set is linear, assume that $f\left(x\right)=\left(\omega \cdot x\right)+b$. With the introduction of the insensitive loss function *ε*, for the nonlinear training set, the data in the input space are mapped into the high-dimensional feature space through a nonlinear mapping Φ, and the regression estimation function is constructed in the space. For the nonlinear case, the estimated function *f*(*x*) is expressed as follows:

(9) $$f\left(x\right)=\langle \omega \cdot \Phi \left(x\right)\rangle +b$$

The optimization problem is

(10) $$\mathrm{Min}\frac{1}{2}{\left\|\omega \right\|}^{2}+C\sum_{i=1}^{l}\left(\varepsilon_{i}+\varepsilon_{i}^{*}\right)\;$$

The constraints are

(11) $$s.t.\left\{\begin{array}{c} y_{i}-\langle \omega^{T}\cdot \Phi \left(x_{i}\right)\rangle -b\leq \varepsilon +\varepsilon_{i}^{*}\\ \langle \omega^{T}\cdot \Phi \left(x_{i}\right)\rangle -b-y_{i}\leq \varepsilon +\varepsilon_{i}^{*}\\ \varepsilon \geq 0\\ \varepsilon_{i}^{*}\geq 0 \end{array}\right.$$

The minimization means minimizing the Vapnik–Chervonenkis (VC) dimension. The model approximates the data points with the accuracy of *ε*. Therefore, the optimization problem reflects the idea of the SVM, and the resulting regression estimation function has good generalization ability. When the constraint condition is unachievable, two slack variables $\varepsilon_{i}$ and $\varepsilon_{i}^{*}$ are introduced. By solving the optimization problem in Equation (11), the approximation function is obtained, as shown in Equation (12):

(12) $$f\left(x\right)=\sum_{i=1}^{l}\left(\varepsilon_{i}+\varepsilon_{i}^{*}\right)K\left(x_{i},x_{j}\right)+b$$

where $K\left(x_{i},x_{j}\right)=\Phi {\left(x_{i}\right)}^{T}\Phi \left(x_{j}\right)$ is called the kernel function.

The statistical learning method of the SVM introduces the kernel function. The nonlinear problem in low-dimensional space is transformed into a linear problem in high-dimensional space. The nonlinear function fitting problem is solved. Compared with other algorithms, the main advantages of the SVM are as follows:

(1) The generalization ability of the SVM is superior to traditional learning methods such as neural networks. The solution of the SVM is transformed into a quadratic programming problem. Therefore, the solution of the SVM is unique and globally optimal;

(2) The small sample problem and the nonlinear problem can be solved by the SVM effectively.

Based on the SVM, the Python language is applied to develop the deflection prediction program for high-strength steel saddle plate forming in this paper. The steps are as follows.

(1) Latin hypercube sampling is used to extract five input vectors of plate length *L*, plate width *B*, curvature radius *R*, plate thickness *t*, and heating line length *l*. The target vector deflection *w* is obtained by numerical calculation;

(2) The data are normalized; 80% of the sample data are extracted as training samples and 20% as test samples;

(3) The Gaussian basis function (RBF) is the type of kernel function. The RBF is mainly used in the case of linear inseparability, which has better application performance for large-sample and small-sample data. The deflection prediction model is established by using the SVM calculation toolbox in the Python compiler;

(4) Design the experimental parameters. The coefficients γ of the kernel function are set to ‘auto’, 0.1, and 1. The penalty parameters *C* are set to 1, 10, 100, and 1000. The loss function *ε* is set to 0.1, 0.05, and 0.01. As shown in Table 4, all possibilities are enumerated. There are 45 sets of experimental parameters in the combination mode;

(5) After the prediction model is established, the test sample set is used to analyze the model, and the experimental parameters with the best prediction effect are obtained.

### 4.2. Sample Sampling and Data Processing

In order to ensure that the sample data are uniformly distributed with equal probability in each variable, the sample is obtained by the Latin hypercube sampling method. Latin hypercube sampling is a hierarchical Monte Carlo sampling method, which is suitable for uniform sampling in multi-dimensional space and suitable for small sample sizes. The sampling range of plate length is 2 m to 5 m. The sampling range of plate width is 1 m to 2 m. The sampling range of curvature radius is 1 m to 5 m. The sampling range of plate thickness is 0.012 m to 0.028 m. The sampling range of heating line length is 0.2 m to 0.4 m. A total of 100 sets of samples are obtained by Latin hypercube sampling. The sampling results are shown in Table 5. The deflection in the table is obtained by numerical calculation.

Because different characteristic parameters generally have different dimensions, the characteristic parameters with larger orders of magnitude have a greater impact on the results, and the influence of the characteristic parameters with smaller orders of magnitude on the results is easily ignored. Therefore, the data are normalized to eliminate the dimension so that each eigenvalue is determined within the same numerical range. The parameters with smaller orders of magnitude are ignored in the regression analysis. The data in the sample data set in Table 5 are normalized. Equation (13) is applied to convert $X_{i}\left(k\right)$ into $X_{i}^{\prime }\left(k\right)$. The normalized results are shown in Table 6.

(13) $$X_{i}^{\prime }\left(k\right)=\frac{X_{i}\left(k\right)-min\left(X_{i}\left(k\right)\right)}{max\left(X_{i}\left(k\right)\right)-min\left(X_{i}\left(k\right)\right)}$$

where $X_{i}\left(k\right)$ is the original sample data; $X_{i}^{\prime }\left(k\right)$ is normalized sample data; $max\left(X_{i}\left(k\right)\right)$ is the maximum value in the original sample data; and $min\left(X_{i}\left(k\right)\right)$ is the minimum value in the original sample data.

### 4.3. Effectiveness Analysis of Intelligent Prediction Model for Deflection

Taking 80% (80 groups) of the samples in Table 6 as the training set and 20% (20 groups) as the test set, the intelligent prediction model for deflection based on the SVM is established by programming. The experimental parameters are obtained from Table 4, and the optimal experimental parameters are determined by analyzing the prediction results. The goodness of fit of $R^{2}$ is often used to evaluate the prediction effect of an SVM. The value of $R^{2}$ is between 0 and 1. The closer its value is to 1, the better the function-fitting effect is. It is verified that the goodness of fit of the 43rd group of samples in Table 4 is 0.997, which is the closest to 1. Therefore, the experimental parameters are set as follows: γ=1, C=10,000, ε=0.01. The deflection prediction results after inverse normalization in the test set are shown in Table 7. Although the relative error of some samples is greater than 5%, it does not mean that the accuracy of the model does not meet the requirements. The standard range of dimensional error for ship double-curved plates in Chinese shipyards is within $3\times 10^{-3}$ m [22]. For example, the value of the relative error of No. 81 in Table 7 is 13.233%. However, the error meets the forming requirements of the saddle plate. Because the value of dimensional error is $0.088\times 10^{-3}$ m, which is much less than $3\times 10^{-3}$ m. To verify the accuracy of the deflection prediction model based on the SVM, the absolute error and quoted error of the test set data are calculated. Quoted error refers to the percentage of absolute error in the full range. Among the 20 sets of data in the test set, the maximum absolute value of absolute error is 9.4 × 10^−5^ m, and the value of quoted error is less than 1%. The reliability of the intelligent prediction model based on the SVM is verified.

### 4.4. Example Verification of Deflection Intelligent Prediction Model

In order to compare the applicability of the intelligent prediction model and the multiple regression prediction model, the example in Table 3 is used to verify the deflection intelligent prediction model. The results are shown in Table 8. The value of the relative error of the example prediction of data interpolation and extrapolation is less than 5%, which is within the acceptable range. The feasibility of the model in the deflection prediction of data extrapolation is verified.

## 5. Conclusions

In this paper, the influence of plate geometric parameters and forming parameters on the deflection of high-strength steel saddle plates was researched. A regression model and intelligent model for deflection prediction of high-strength steel saddle plates were established in this research. The conclusions are as follows.

1. The deflection is positively correlated with the size of the plate length, curvature radius, and heating line length. The deflection is negatively correlated with the size of plate thickness and heating velocity.
2. There is a linear relationship between the deflection, curvature radius, and heating line length. There is a nonlinear relationship between deflection, plate length, width, and thickness. The independent variables of the nonlinear relationship are linearized by using the perfect square formula.
3. The deflection prediction model of multiple linear regression was established based on the relationship between deflection and influence factors. The values of relative errors, for example, for interpolation, are less than 5%, which verifies the reliability of the multiple regression model.
4. The intelligent prediction model based on an SVM was established. The values of reference errors, for example, for extrapolation, are less than 5% with optimal experimental parameters. The intelligent prediction model can be applied to the deflection prediction of marine saddle plate forming. This research can also provide data support for the automatic forming of marine saddle plates.

## Figures and Tables

**Table 1:** Chemical composition of EH36 steel.

Chemical Element	C	Si	Mn	P	S	Cu	Ni	Cr	Nb	Ti	Als
Mass fraction (%)	0.13	0.32	1.11	0.016	0.0006	0.24	0.24	0.16	0.026	0.013	0.04

**Table 2:** The maximum temperature of the plate surface at the minimum heating velocity.

Plate Thickness ($10^{-3}$ m)	Minimum Heating Velocity ($10^{-3}$ m/s)	The Maximum Temperature (°C)	Relative Error
8	3.770	659.311	0.97%
12	2.740	643.622	−0.82%
16	2.185	645.453	−0.70%
20	1.833	648.514	−0.23%
24	1.588	650.915	0.14%
28	1.407	652.254	0.35%
32	1.266	653.405	0.52%

**Table 3:** The example verification of multiple regression model.

*L* (m)	*B* (m)	*R* (m)	*t* (m)	*l* (m)	Deflection Obtained by FEM ($10^{-3}$ m)	The Calculated Deflection of Equation (3) ($10^{-3}$ m)	Absolute Error ($10^{-3}$ m)	Relative Error	Example Location
3.3	1.6	4.5	0.008	0.3	4.369	4.218	−0.152	−3.474%	Interpolation
2.8	1.5	4.0	0.012	0.3	2.576	2.578	0.002	−0.078%	Interpolation
3.7	1.0	3.6	0.016	0.2	7.608	7.566	−0.042	−0.052%	Interpolation
3.0	1.6	2.1	0.008	0.4	2.606	2.716	0.109	4.221%	Interpolation
5.1	1.5	5.0	0.012	0.3	11.075	10.266	−0.809	−7.309%	Extrapolation
5.2	1.5	5.0	0.012	0.3	11.849	10.693	−1.156	−9.755%	Extrapolation
5.5	1.5	5.5	0.016	0.3	14.461	12.039	−2.422	16.751%	Extrapolation

**Table 4:** Experimental parameter design.

No.	Kernel Function Coefficient *γ*	Penalty Parameter *C*	Loss Function *ε*
1	‘auto’	1	0.01
2	‘auto’	1	0.05
3	‘auto’	1	0.1
4	‘auto’	10	0.01
5	‘auto’	10	0.05
⋯	⋯	⋯	⋯
43	1	10,000	0.01
44	1	10,000	0.05
45	1	10,000	0.1

**Table 5:** Sample results obtained by Latin hypercube sampling.

No.	Plate Length (m)	Plate Width (m)	Curvature Radius (m)	Plate Thickness (m)	Heating Line Length (m)	Deflection ($10^{-3}$ m)
1	4.8	1.7	4.8	0.020	0.3	4.884
2	4.6	1.2	1.5	0.016	0.3	1.910
3	4.3	1.8	3.6	0.016	0.3	1.878
4	3.3	1.6	4.5	0.008	0.3	4.369
5	4.3	2.4	4.5	0.012	0.4	1.554
⋯	⋯	⋯	⋯	⋯	⋯	⋯
98	3.5	1.1	4.7	0.028	0.2	4.598
99	3.8	2	3.7	0.024	0.2	1.141
100	2.9	1.2	3.7	0.028	0.3	2.136

**Table 6:** Normalized sample data for deflection prediction.

No.	Normalized Parameters					
	Plate Length	Plate Width	Curvature Radius	Plate Length	Heating Line Length	Deflection
1	0.931	0.467	0.943	0.600	0.500	0.487
2	0.862	0.133	0.000	0.400	0.500	0.160
3	0.759	0.533	0.600	0.400	0.500	0.157
4	0.414	0.400	0.857	0.000	0.500	0.430
5	0.759	0.933	0.857	0.200	1.000	0.121
⋯	⋯	⋯	⋯	⋯	⋯	⋯
97	0.655	0.333	0.514	0.800	0.500	0.201
98	0.483	0.067	0.914	1.000	0.000	0.455
99	0.586	0.667	0.629	0.800	0.000	0.076
100	0.276	0.133	0.629	1.000	0.500	0.185

**Table 7:** Prediction results and error analysis of test set.

No.	Deflection Obtained by FEM ($10^{-3}$ m)	Prediction Deflection ($10^{-3}$ m)	Absolute Error ($10^{-3}$ m)	Relative Error	Quoted Error
81	0.665	0.577	0.088	13.233%	0.919%
82	0.764	0.853	−0.090	−11.780%	−0.941%
83	2.807	2.900	−0.093	−3.313%	−0.977%
84	0.598	0.692	−0.094	−15.719%	−0.984%
⋯	⋯	⋯	⋯		⋯
97	2.278	2.264	0.014	0.614%	0.145%
98	4.598	4.638	−0.040	−0.870%	−0.420%
99	1.142	1.234	−0.093	−8.14%	−0.971%
100	2.136	2.042	0.093	4.354%	0.975%

**Table 8:** Example verification of intelligent prediction model for deflection.

*L* (m)	*B* (m)	*R* (m)	*t* (m)	*l* (m)	Deflection Obtained by FEM ($10^{-3}$ m)	Prediction Deflection ($10^{-3}$ m)	Absolute Error ($10^{-3}m$ )	Relative Error	Example Location
3.3	1.6	4.5	0.008	0.3	4.369	4.280	−0.090	−2.050%	Interpolation
2.8	1.5	4.0	0.012	0.3	2.576	2.666	0.090	3.484%	Interpolation
3.7	1.0	3.6	0.016	0.2	7.608	7.515	−0.092	−1.212%	Interpolation
3.0	1.6	2.1	0.008	0.4	2.606	2.515	−0.091	−3.52%	Interpolation
5.1	1.5	5.0	0.012	0.3	11.075	11.193	0.118	1.067%	Extrapolation
5.2	1.5	5.0	0.012	0.3	11.849	11.738	−0.110	0.935%	Extrapolation

## Data Availability

Not applicable.
